# Alternating current line-filter based on electrochemical capacitor utilizing template-patterned graphene

**DOI:** 10.1038/srep10983

**Published:** 2015-06-17

**Authors:** Zhenkun Wu, Liyi Li, Ziyin Lin, Bo Song, Zhuo Li, Kyoung-Sik Moon, Ching-Ping Wong, Shu-Lin Bai

**Affiliations:** 1 Department of Materials Science and Engineering, CAPT/HEDPS/LTCS, Key Laboratory of Polymer Chemistry and Physics of Ministry of Education, College of Engineering, Peking University, Beijing 100871, China; 2 School of Material Science and Engineering, Georgia Institute of Technology, Atlanta, GA 30332-0245, USA

## Abstract

Aluminum electrolytic capacitors (AECs) are widely used for alternating current (ac) line-filtering. However, their bulky size is becoming more and more incompatible with the rapid development of portable electronics. Here we report a scalable process to fabricate miniaturized graphene-based ac line-filters on flexible substrates at room temperature. In this work, graphene oxide (GO) is reduced by patterned metal interdigits at room temperature and used directly as the electrode material. The as-fabricated device shows a phase angle of −75.4° at 120 Hz with a specific capacitance of 316 µF/cm^2^ and a RC time constant of 0.35 ms. In addition, it retains 97.2% of the initial capacitance after 10000 charge/discharge cycles. These outstanding performance characteristics of our device demonstrate its promising to replace the conventional AECs for ac line filtering.

Alternating current (ac) line-filters are used to smooth the leftover ac ripples on direct current (dc) voltage buses for most line-powered devices. To effectively filter out the ac ripples on dc input, capacitors with large capacitance are required in ac line-filtering devices. Currently, aluminum electrolytic capacitors (AECs) are widely used for this purpose due to their relatively lower fabrication cost and higher specific capacitance compared to thin film capacitors. However, they are still the largest component in many electronic systems. Their bulky volume is becoming more and more incompatible with the rapid development of miniaturized portable and wearable electronics. As a result, it is of great interest to develop a capacitor with competitive performance yet much smaller volume. Electrochemical capacitors, also known as supercapacitors, may help to alleviate the volumetric problem of the conventional ac filters due to their extremely large capacitance density. Their specific capacitances are generally several orders of magnitude higher than other types of capacitors^1^. Thus it will require much less space to produce the designed capacitance. Different electrode materials are extensively studied, including carbon derivatives^2^,^3^,^4^, conducting polymers^5^,^6^, metal oxides^7^,^8^ and their hybrids^9^,^10^. Unfortunately, their frequency response is still unsatisfactory and requires further improvement. For example, the phase angle of a typical supercapacitor approaches zero degree at 120 Hz for most supercapacitors, which is far below the required –90° for ac line-filtering. In principal, supercapacitors based on electrical double-layer capacitance (EDLC) are similar to AECs in which they both adopt physical storage mechanism. They are potentially capable of replacing the traditional AECs if their frequency response can be greatly improved. In this sense graphene and carbon nanotubes are very promising due to their extremely high specific surface area and excellent electrical conductivity. Recently devices based on these carbon allotropes have been designed and fabricated with comparable rate performance to those of AECs^11^,^12^,^13^,^14^,^15^,^16^,^17^. For example, Miller *et al.* used a radio frequency plasma enhanced chemical vapor deposition method (PECVD) to fabricate vertically oriented graphene (VG) sheets as the active material and reported a phase angle of −82° at 120 Hz^13^. Lin *et al.* designed interdigital micro-supercapacitors with graphene/vertically aligned carbon nanotube arrays (VACNT), which was grown by a conventional CVD approach, and achieved a phase angle of −81.5° at 120 Hz^14^. Sheng *et al.* electrochemically reduced graphene oxide (GO) on a gold foil and reported a phase angle of −84° at 120 Hz^15^. However, most of these methods are not suitable for large scale production. In addition, the high processing temperature is incompatible with flexible substrate that is critical for wearable electronics. Here we designed a novel wet chemical method to fabricate a graphene based ac line-filter. The as-fabricated device shows a phase angle of −75.4° at 120 Hz with a specific capacitance of 316 μF/cm^2^ and a RC time constant of 0.35 ms. In addition, it retains 97.2% of the initial capacity after 10000 galvanostatic charge/discharge (CD) cycles.

## Results

**Figure 1:**
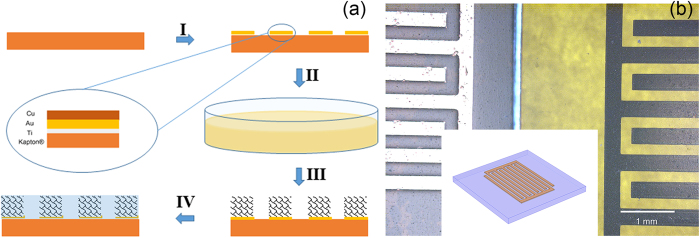
a) Illustration of the fabrication process. I, photolithography process; II, GO reduction; III, washing and cleaning; IV, electrolyte infiltration. The inset represents the cross-section sketch of the metal interdigit. (**b**) Optical images of the device before (left) and after (right) rGO assembling and air-drying. Inset: the illustration of the substrate patterned with metal interdigit.

The fabrication procedure is illustrated in Fig. 1(a). First, a metal inter-digital pattern is fabricated on a polyimide substrate. The as-prepared metal interdigit consists of three layers of metals: on top is a 200 nm Cu layer acting as the reducing agent during the GO reduction and assembly process; beneath Cu is a 200 nm Au layer, which serves as the current collector after the Cu layer on top is oxidized and removed; at the bottom is a 10 nm Ti layer used to improve the adhesion between the metal interdigit and the polyimide substrate. Then the substrate patterned metal interdigit is soaked in the GO solution for certain time^18^,^19^, during which the GO sheets are simultaneously reduced by the Cu layer on top and assembled onto the metal interdigit. After that the sample is mildly washed with deionized water and soaked in 1 M sulfuric acid for 3 hours before electrochemical tests. The aqueous sulfuric acid serves as the electrolyte for our device.

Active metals such as Cu, Zn and Ni have been used to reduce GO because of their lower redox potential compared to that of GO^20^,^21^,^22^. When the metal substrate is immersed into a GO solution, some of the GO sheets will diffuse onto the metal surface and be reduced *in situ*. Since the as-reduced rGO sheets are electrically conductive yet insoluble in water, they will remain on the substrate and serve as an electron conducting path, leading to a continuous reduction process. This *in situ* reduction and assembling mechanism offers a convenient way to pattern rGO. Figure 1(b) presents optical images of the as-fabricated metal interdigit with and without rGO assembling. The shiny golden interdigit turns into black stripes with the rGO on top. In addition, the interdigit fingers remain sharp and straight without any GO residues in between, which avoids the risk of short-circuit failures during operation. The inset is the sketch of the metal interdigit pattern. Here Cu is chosen as the reducing agent mainly for two reasons: (1) Cu produces the best rGO in terms of the carbon-to-oxygen ratio compared to other metals like Zn and Fe, even though Cu is usually considered less reactive^20^; (2) the photo-lithography technique for patterning of Cu is very mature.

Our innovative approach provides many advantages. Firstly, it is prepared at room temperature, which offers great flexibility on substrate selections. For example, flexible polymer substrates such as polyimide or polyethylene terephthalate (PET), which cannot stand high temperature operation, can be employed. It thus enables the ability to be integrated into wearable electronics. Secondly, it utilizes an interdigital structure that avoids the need of a bulky separator membrane and greatly eases the fabrication process. Moreover, our approach is readily scalable. All the steps involved, such as the photolithography step and the solution processing, are well established and mature. As a result, it reduces the fabrication cost significantly compared to those CVD methods.

**Figure 2:**
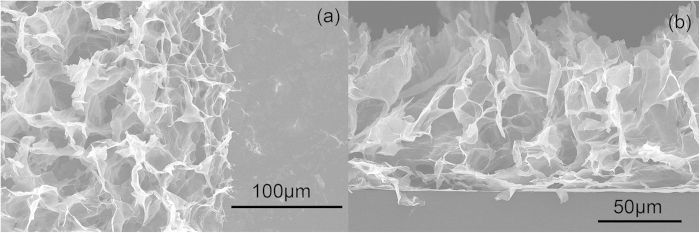
a) Top-view and (b) side-view of a free-dried rGO aerogel on a metal interdigit finger.

**Figure 3:**
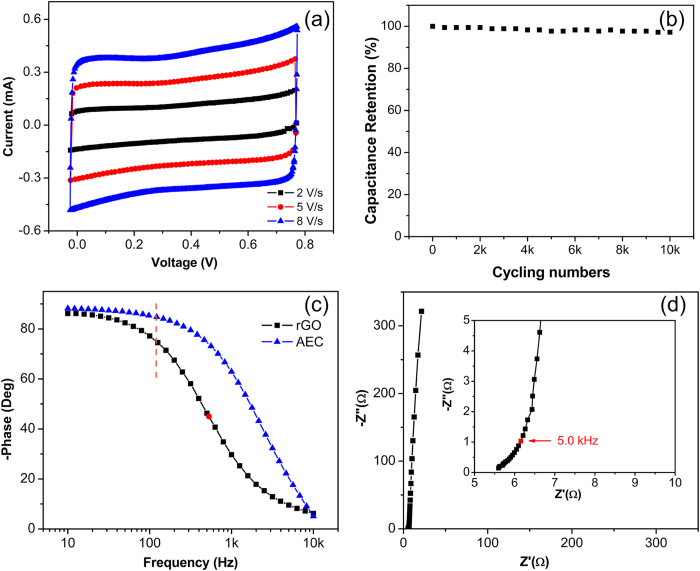
**a**) CV curves of the device at scan rates of 2, 5 and 8 V/s, respectively.(**b**) Stability performance measured with CD cycling at a current density of 1.3 mA/cm^2^. (**c**) Bode phase diagram of our device and AEC. (**d**) AC impedance spectrum of our device. The inset shows the high frequency region.

Figure 2(a,b) shows the top and cross-section scanning electron microscopic (SEM) images of the freeze-dried rGO aerogel assembled on the metal interdigit of a silicon substrate. The as-reduced rGO sheets form a three-dimensional interconnected structure that attaches onto the metal interdigit instead of diffusing away. The sharp edges clearly maintain the original metal interdigital design and illustrate the conformal coating mechanism. Electrochemical tests were carried out to test the performance. Figure 3(a) shows the cyclic voltammetry (CV) curves at scan rates of 2, 5 and 8 V/s, which are approximately two orders of magnitude higher than those for conventional supercapacitors. The rectangular CV curves at high scan rates imply small series resistance and efficient EDLC attribute of the as-prepared rGO hydrogel electrode^23^. Figure 3(b) shows the cycling performance of the device. The capacitance retention is 97.2% after 10000 times of CD cycles at a current density of 0.58 mA/cm^2^. The good cycling performance of our device is highly desirable in real applications.

## Discussion

The fast ion transport as well as the excellent stability is highly desirable for ac line-filtering. To justify the feasibility of our device, electrochemical impedance spectroscopy (EIS) is carried out from 10 Hz to 100 kHz with a 10 mV sinusoidal voltage input. The impedance phase angle (also known as Bode phase) for ideal capacitors is −90° regardless of the frequency. In real-life applications, the phase angle approaches −90° at low frequencies and then decreases as frequency further increases. The value at 120 Hz is usually used as a figure of merit to estimate the filtering efficacy. Figure 3(c) presents the impedance phase angle diagram for our as-fabricated ac line-filter. The phase angle maintains above −80° at frequency below 79 Hz and reaches −75.4° at 120 Hz. The frequency at −45°, usually used for comparison, reaches 529 Hz in our case. These parameters are better than those high-power supercapacitors fabricated with laser-scribed graphene (LSG)^17^, onion-like carbon (OLC)^24^ and carbon nanotubes (CNT)^25^,^26^. The superior performance is caused by the high-quality electrical conductive rGO electrodes that avoid distributed charge storage. Our rGO electrode achieves a high carbon-to-oxygen ratio of 6.8 for from the quantitative XPS survey, much higher than those reported with metal^20^,^21^ and thermal reduction methods^27^,^28^. Figure 3(d) exhibits a nearly vertical impedance spectrum at low frequency region for the as-fabricated ac line-filter, which indicates almost pure capacitive behavior^3^. Typically a semi-circle is found in activated carbon^3^,^29^ and rGO^2^,^30^ based supercapacitors at the high frequency region of the Nyquist plot; this semi-circle is caused by the capacitive coupling between the electrode and the current collector. In contrast, no semi-circle appears at the high frequency region for our device (Fig. 3(d), inset), suggesting excellent electrical connection at the interface between the rGO electrode and the gold current collector.

**Figure 4:**
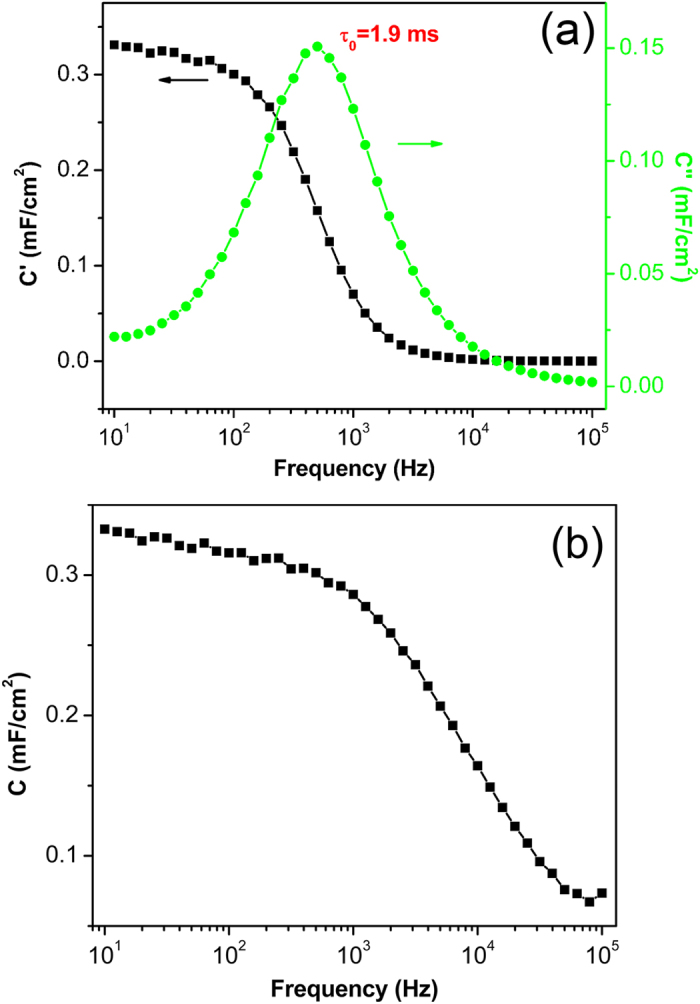
(a) The variation of real and imaginary part of capacitance versus frequency.**b**) Capacitance versus frequency.

The outstanding performance is further supported with the ultra-small relaxation time constant (τ_0_). τ_0_ reflects the minimum time needed to discharge all the energy from the device with an efficiency of greater than 50%, which can be obtained from the frequency at a maximum imaginary capacitance (C”)^14^,^15^,^31^. Figure 4(a) shows an extremely small τ_0_ of 1.9 ms for the as-fabricated ac line-filter. Meanwhile, a series RC circuit model, also known as Randles circuit model, is employed to simulate the capacitive and resistive components of the device. The specific areal capacitance is calculated based on the equation of 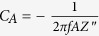, in which *f* is the frequency, *A* is the area of the device and *Z”* is the imaginary part of the impedance^31^. Figure 4(b) presents the frequency response specific areal capacitance, which reaches 316 μF/cm^2^ at 120 Hz and maintains capacitive behavior up to 10^4^ Hz. The resistance at 120 Hz is 7.5 Ω, which corresponds to a RC time constant τ_RC_ of 0.35 ms considering an electrode area of 14.88 mm^2^ of the device. This value is comparable to those reported with vertical graphene EDLC (0.20 ms)^13^, graphene/VACNT hybrids (0.20 ~ 0.40 ms)^14^ and electrochemically reduced GO (1.35 ms)^15^. It is also significantly smaller than the required 8.3 ms for 120 Hz filtering.

A detailed comparison of performance with other ac line-filters is listed in Table 1. Our device exhibits very promising phase angle at 120 Hz, which is comparable to most of those reported in literature and slightly lower than that of electrochemically reduced GO and commercial AEC. It also possesses excellent time relaxation constants and RC time constant, much shorter than the required 8.3 ms for ac line-filtering. In addition, its specific areal capacitance is one of the best among those reported with phase angle better than −70° at 120 Hz, which is beneficial for miniaturization. These attributes make it very promising in real-life applications.

In conclusion, we have designed a scalable method to manufacture ac line-filter based on patterned rGO. The procedure is solution based and carried out at room temperature, which makes it compatible with flexible substrates. The rate performance, areal capacitance and RC time constant of our device are competitive among all the reported ac line-filtering devices. The high areal capacitance of the device benefits significantly to the miniaturization of whole electronic system. The excellent rate performance, scalable room temperature manufacturing process and compatibility with flexible substrates make it suitable for ac line-filtering in the field of wearable electronics.

## Methods

GO was prepared with a modified Hummers’ method from natural graphite (see Supplementary Information for details)^18^,^19^. A metal inter-digital pattern (finger: length 4 mm, width 0.3 mm; gap between fingers: 0.2 mm; total dimension: 4.8 × 4.8 mm^2^) was fabricated on a polyimide substrate (125 μm thick, Kapton^®^, Dupont, USA) by standard photolithography and electron-beam evaporation. The pattern was made up of three layers of metals with different thickness, namely Ti (10 nm) at the bottom, Au (200 nm) in the middle and Cu (200 nm) on the top. The metal interdigit-patterned substrate was then soaked in the GO solution (1 mg/mL, 50 mL) for 12 hours. After that the sample was mildly washed with deionized water and soaked in 1 M sulfuric acid for three hours before electrochemical tests.

Morphology of rGO hydrogel was observed with SEM (Hitachi SEM SU8010). X-ray photoelectron spectrum (XPS) was obtained using a Thermo K-Alpha XPS. Raman spectra were obtained with LabRAM ARAMIS, HORIBA JOBIN YVON with 532 nm laser used as the excitation source. Fourier transform infrared spectrum (FTIR) was measured with FTIR spectrometer (Nicolet, Magna IR560). CV, CD and EIS were carried out with a Versastat 2-channel system (Princeton Applied Research). Specific areal capacitance was derived from the ac impedance spectrum.

## Additional Information

**How to cite this article**: Wu, Z. *et al.* Alternating current line-filter based on electrochemical capacitor utilizing template-patterned graphene. *Sci. Rep.* **5**, 10983; doi: 10.1038/srep10983 (2015).

## Supplementary Material

Supplementary Information

## Figures and Tables

**Table 1:** Comparison of electrochemical performance of ac line-filters using different electrode materials.

**Material**	**Substrate**	**-Phase at 120 Hz (°)**	**τ**_**RC**_ **(ms)**	**τ**_**0**_ **(ms)**	**Capacitance (mF/cm**^2^)
this work	F^a^	75.4	0.35	1.9	0.32
AEC	N^a^	84.8	0.11	0.46	N/A
VG^13^	N	82	~ 0.2	N/A	0.0875
Graphene/VACNT^14^	N	73.4 ~ 81.5	0.2 ~ 0.4	0.82 ~ 2.62	0.23 ~ 0.66
rGO^15^	F	79.5 ~ 85	1.35	0.17 ~ 1.0	0.283
LSG^17^	F	<30	N/A	19	N/A
OLC^24^	F	N/A	N/A	26	N/A

^a^F and N designate that the substrate can and cannot be flexible, respectively.
