# Supplementary material for: Alternating current line-filter based on electrochemical capacitor utilizing template-patterned graphene
Source: Sci Rep. 2015 Jun 17;5:10983. doi: 10.1038/srep10983 (PMC4470326; doi:10.1038/srep10983)
Supplement: Supplementary Information [file srep10983-s1.pdf]

Supplementary Information

Alternating current line-filter based on electrochemical capacitor utilizing template-patterned graphene

Zhenkun Wu,[1,2*] Liyi Li,[1,*] Ziyin Lin,[1] Bo Song,[1] Zhuo Li,[1] Kyoung-Sik Moon,[1] Shu-Lin Bai,[2] Ching-Ping Wong[1]

[1]*School of Material Science and Engineering, Georgia Institute of Technology, Atlanta, GA 30332-0245, USA*

[2]*Department of Materials Science and Engineering, College of Engineering, Peking University, Beijing 100871, China*

1. Supplementary materials and methods

Synthesis of Graphene Oxide (GO)

GO was prepared according to modified Hummers method[1]. $NaNO_3$ (4 g) was stirred and dissolved in H_2SO_4 (98 wt%, 200 mL) in ice bath. Graphite powder (Asbury 230U, 4 g) was slowly added into the mixture. After that $KMnO_4$ (18 g) was gradually added below 8 ^{0}C. The mixture was stirred for 2 hours in ice bath 30 minutes at 35 ^{0}C water bath successively. Then deionized water (500 mL) was added slowly into the solution while maintaining the temperature below 80 ^{0}C. In the end H_2O_2 (30% w/w in H_2O, 50 mL) was added and the color turned into golden yellow. The GO obtained was washed with HCl (1 M, 1 L) twice and deionized water several times until the pH reached 6.

Fabrication of Au/Cu inter-digital pattern

First a 1.6 µm thick layer of positive resist (Microposit S1813, hipley, USA) was spun cast on the polyimide substrate (125 µm Kapton®, Dupont, USA). Then it was exposed into a 10-finger inter-digital design with finger width of 300 µm, finger gap 200 µm and total dimension 4.8×4.8 mm^2 with a mask aligner (Karl Suss MA6 Mask Aligner, Garching, Germany) applying 405 nm UV light. The exposed samples were developed (Microposit MF 315, Shipley, USA) and cleaned by oxygen plasma in a reactive ion etching (RIE) tool (Plasma Therm Inc., USA) to remove any resist residue in exposed areas. Thin layers of Ti (10 nm), Au (200 nm) and Cu (200 nm) were electron beam deposited onto the substrate successively (pressure of 3×10^{-6} Torr, CVC Product Inc., USA). In the end, the photoresist was lifted off with 5 minutes of sonication in acetone and dried by N_2 flow.

2. Characterization

The successful reduction of GO by Cu interdigit is confirmed with X-ray photoelectron spectrum (XPS, Thermo K-Alpha), Raman spectroscopy (LabRAM ARAMIS, HORIBA JOBIN YVON) and Fourier transform infrared spectrum (FTIR, Nicolet, Magna IR 560).

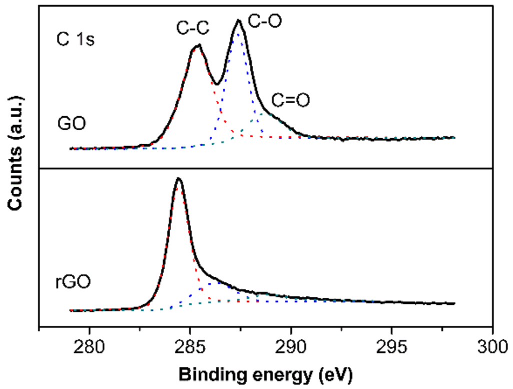

Figure S1. Deconvoluted C1s XPS spectra of GO and rGO reduced with Cu interdigit. The peak intensity corresponding to C-O and C=O bonds are significantly reduced after the reduction process.

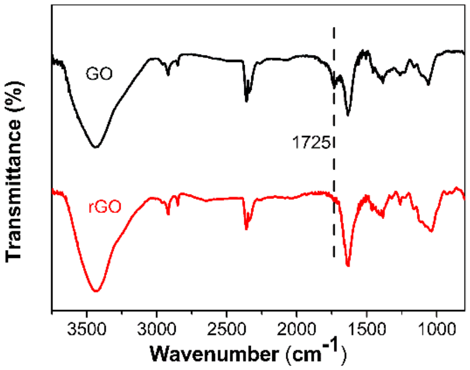

Figure S2. FTIR spectra of GO and rGO. The peak at 1725 cm^{-1} corresponding to carbonyl group disappears after the reaction, further validating the reduction of GO[2].

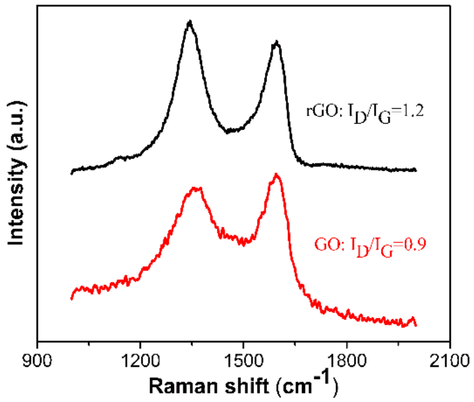

Figure S3. Raman spectra of GO and rGO. The I_D/I_G ratio is increased from 0.9 to 1.2, probably caused by the formation of smaller sp2 domains during reduction[3].

1 W. S. Hummers Jr and R. E. Offeman, Journal of the American Chemical Society **80**, 1339 (1958).

2 Z. Lin, Y. Liu, Y. Yao, O. J. Hildreth, Z. Li, K. Moon, and C.-p. Wong, J. Phys. Chem. C **115**, 7120 (2011); T. Szabó, O. Berkesi, P. Forgó, K. Josepovits, Y. Sanakis, D. Petridis, and I. Dékány, Chem. Mater. **18**, 2740 (2006).

3 H. Wang, J. T. Robinson, X. Li, and H. Dai, J. Am. Chem. Soc. **131**, 9910 (2009).
